# *ent*-5α,3,15-Dioxodolabr-4(18)-ene-16,18-diol

**DOI:** 10.1107/S1600536810042078

**Published:** 2010-10-23

**Authors:** Hoong-Kun Fun, Charoen Pakathirathien, Chatchanok Karalai, Suchada Chantrapromma

**Affiliations:** a X-ray Crystallography Unit, School of Physics, Universiti Sains Malaysia, 11800 USM, Penang, Malaysia; b Department of Chemistry, Faculty of Science, Prince of Songkla University, Hat-Yai, Songkhla 90112, Thailand; c Crystal Materials Research Unit, Department of Chemistry, Faculty of Science, Prince of Songkla University, Hat-Yai, Songkhla 90112, Thailand

## Abstract

The title compound, C_20_H_30_O_4_, is a dolabrane diterpenoid isolated from *Ceriops tagal*, in which one of the three fused cyclo­hexane rings adopts a half-chair conformation and the other two are in the standard chair conformations. The hy­droxy­methyl­idene substituent is attached to the half-chair cyclo­hexane. An intra­molecular O—H⋯O hydrogen bond generate an *S*(6) ring motif. In the crystal, mol­ecules are arranged into screw chains along the [001] direction. The crystal is stabilized by O—H⋯O hydrogen bonds and weaker C—H⋯O inter­actions.

## Related literature

For hydrogen-bond motifs, see: Bernstein *et al.* (1995 ▶). For bond-length data, see: Allen *et al.* (1987 ▶). For ring conformations, see: Cremer & Pople (1975 ▶). For background to diterpenoids, see, for example: Hu *et al.* (2010 ▶); Zhang *et al.* (2005 ▶). For related structures, see: Chantrapromma *et al.* (2007 ▶); Fun *et al.* (2006 ▶). For the stability of the temperature controller used in the data collection, see Cosier & Glazer, (1986 ▶).
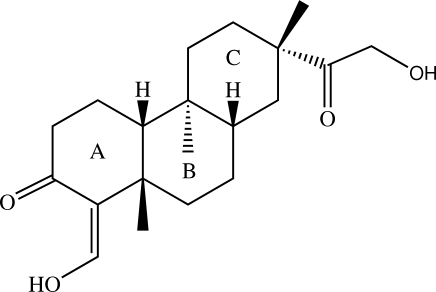


## Experimental

### 

#### Crystal data

C_20_H_30_O_4_
*M* *_r_* = 334.44
Orthorhombic, 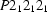

*a* = 7.9633 (3) Å
*b* = 10.7166 (4) Å
*c* = 20.8338 (7) Å
*V* = 1777.95 (11) Å^3^
*Z* = 4
Mo *K*α radiation
μ = 0.09 mm^−1^
*T* = 100 K
0.58 × 0.51 × 0.10 mm

#### Data collection

Bruker APEXII CCD area-detector diffractometer
Absorption correction: multi-scan (*SADABS*; Bruker, 2005 ▶) *T*_min_ = 0.952, *T*_max_ = 0.992
20568 measured reflections
2691 independent reflections
2084 reflections with *I* > 2σ(*I*)
*R*_int_ = 0.030

#### Refinement

*R*[*F* ^2^ > 2σ(*F* ^2^)] = 0.052
*wR*(*F* ^2^) = 0.157
*S* = 1.09
2691 reflections
220 parameters
H-atom parameters constrained
Δρ_max_ = 0.31 e Å^−3^
Δρ_min_ = −0.45 e Å^−3^

### 

Data collection: *APEX2* (Bruker, 2005 ▶); cell refinement: *SAINT* (Bruker, 2005 ▶); data reduction: *SAINT*; program(s) used to solve structure: *SHELXTL* (Sheldrick, 2008 ▶); program(s) used to refine structure: *SHELXTL*; molecular graphics: *SHELXTL*; software used to prepare material for publication: *SHELXTL* and *PLATON* (Spek, 2009 ▶).

## Supplementary Material

Crystal structure: contains datablocks global, I. DOI: 10.1107/S1600536810042078/fj2353sup1.cif

Structure factors: contains datablocks I. DOI: 10.1107/S1600536810042078/fj2353Isup2.hkl

Additional supplementary materials:  crystallographic information; 3D view; checkCIF report

## Figures and Tables

**Table 1:** Hydrogen-bond geometry (Å, °)

*D*—H⋯*A*	*D*—H	H⋯*A*	*D*⋯*A*	*D*—H⋯*A*
O2—H1*O*2⋯O1	0.82	1.69	2.424 (4)	148
O4—H1*O*4⋯O1^i^	0.82	2.07	2.841 (3)	156
C1—H1*B*⋯O2^ii^	0.97	2.48	3.368 (5)	152
C12—H12*A*⋯O3	0.97	2.41	2.799 (4)	103
C17—H17*A*⋯O4^iii^	0.96	2.53	3.460 (5)	164

Symmetry codes: (i) 
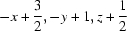
; (ii) 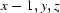; (iii) 
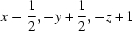
.

**Fig. 1.**
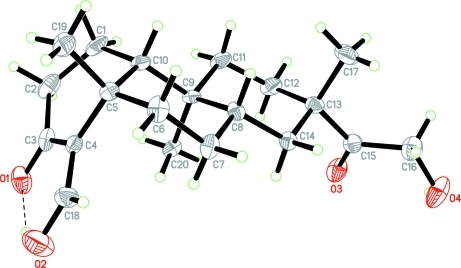
The structure of (I), showing 50% probability displacement ellipsoids and the atom-numbering scheme. Hydrogen bonds was drawn as dash line.

**Fig. 2.**
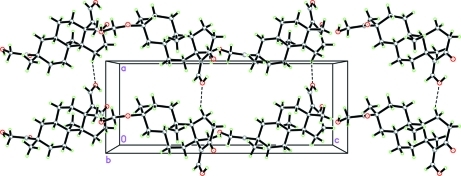
The crystal packing of (I) viewed along the b axis, showing one dimensional chains along the [0 0 1] direction. Hydrogen bonds were shown as dashed lines.

## supplementary crystallographic information

### Comment

*Ceriops tagal* (Perr.) C. B. Robinson is a mangrove plant belonging to the Rhizophoraceae family. Diterpenoids and triterpenoids are the main secondary metabolites of *C. tagal* (Chantrapromma *et al.*, 2007; Hu *et al.*, 2010; Zhang *et al.*, 2005). During the course of our studies on the chemical constituents and bioactive compounds from Thai medicinal plants, the title dolabrane diterpenoid compound (I), which is known as Tagalsin S (Hu *et al.*, 2010), was isolated from the the stem barks of *C. tagal*. We have also previously reported the crystal structures of two diterpenoid compounds isolated from the same plant (Chantrapromma *et al.*, 2007; Fun *et al.*, 2006). We herein report the crystal structure of (I).

The molecule of the title compound contains a fused three-ring system *A/B/C* (Fig. 1). The *A/B* ring junction is *cis*-fused and *B/C* is *trans*-fused. The cyclohexane ring *A* adopts half-chair conformation with puckering parameters Q = 0.539 (3) Å, θ = 111.0 (3)° and φ = 92.5 (4)°, rings *B* and *C* are in standard chair conformations (Cremer & Pople 1975). The hydroxylmethylidine substituent is planarly attached to cyclohexane ring *A* at atom C4 as indicated by the torsion angle C3—C4—C18—O2 of 4.4 (5)° and the bond angles around atom C4 are indicative of *sp*^2^ hybridization for this atom. The orientations of the carbonyl and alcohol substituent groups at atom C13 are described by the torsion angles C13–C15—C16—O4 = 166.3 (3)° and O3–C15–C16–O4 = -11.1 (5)°. Intramolecular O2—H1O2···O1 hydrogen bond (Table 1) generates S(6) ring motif (Fig. 1) (Bernstein *et al.*, 1995). The bond distances are of normal values (Allen *et al.*, 1987) and are comparable with the related structures (Chantrapromma *et al.*, 2007; Fun *et al.*, 2006).

In the crystal structure (Fig. 2), the molecules are arranged into screw chains along the [0 0 1] direction and the adjacent chains are further linked by weak C—H···O interactions (Table 1). The crystal packing of (I) is stabilized by intermolecular O—H···O hydrogen bonds and weak C—H···O interactions (Fig. 2 and Table 1).

### Experimental

The air-dried and crushed stem barks of *C. tagal* (4.8 kg) were extracted with methylene chloride and then concentrated *in vacuo* to give a residue (17.4 g). This residue was subjected to quick column chromatography over silica gel using solvents of increasing polarity from hexane through 50% acetone/hexane. The eluates were collected and combined, based on TLC, to give 20 fractions (F1—F20). Fraction F14 was further purified by repeated quick column chromatography with CH_2_Cl_2_/acetone (9:1 *v*/*v*) yielding title compound (30.4 mg). Colorless block-shaped single crystals of the title compound suitable for *x*-ray structure determination were recrystallized from hexane/CH_2_Cl_2_ (1:1, *v*/*v*) after several days, Mp. 395–396 K.

### Refinement

All H atoms were placed in calculated positions with d(O—H) = 0.82 Å and d(C—H) = 0.93 Å for aromatic and CH, 0.97 for CH_2_ and 0.96 Å for CH_3_ atoms. The *U*_iso_ values were constrained to be 1.5*U*_eq_ of the carrier atom for hydroxy and methyl H atoms and 1.2*U*_eq_ for the remaining H atoms. A rotating group model was used for the methyl groups. The highest residual electron density peak is located at 0.18 Å from H1B and the deepest hole is located at 0.50 Å from O2. A total of 2024 Friedel pairs were merged before final refinement as there is no large anomalous dispersion for the determination of the absolute configuration.

### Crystal data

**Table:**

C_20_H_30_O_4_	*D*_x_ = 1.249 Mg m^−^^3^
*M**_r_* = 334.44	Melting point = 495–496 K
Orthorhombic, *P*2_1_2_1_2_1_	Mo *K*α radiation, λ = 0.71073 Å
Hall symbol: P 2ac 2ab	Cell parameters from 2691 reflections
*a* = 7.9633 (3) Å	θ = 2.0–29.0°
*b* = 10.7166 (4) Å	µ = 0.09 mm^−^^1^
*c* = 20.8338 (7) Å	*T* = 100 K
*V* = 1777.95 (11) Å^3^	Plate, colourless
*Z* = 4	0.58 × 0.51 × 0.10 mm
*F*(000) = 728	

### Data collection

**Table:**

Bruker APEXII CCD area-detector diffractometer	2691 independent reflections
Radiation source: sealed tube	2084 reflections with *I* > 2σ(*I*)
graphite	*R*_int_ = 0.030
φ and ω scans	θ_max_ = 29.0°, θ_min_ = 2.0°
Absorption correction: multi-scan (*SADABS*; Bruker, 2005)	*h* = −10→10
*T*_min_ = 0.952, *T*_max_ = 0.992	*k* = −14→10
20568 measured reflections	*l* = −24→28

### Refinement

**Table:**

Refinement on *F*^2^	Primary atom site location: structure-invariant direct methods
Least-squares matrix: full	Secondary atom site location: difference Fourier map
*R*[*F*^2^ > 2σ(*F*^2^)] = 0.052	Hydrogen site location: inferred from neighbouring sites
*wR*(*F*^2^) = 0.157	H-atom parameters constrained
*S* = 1.09	*w* = 1/[σ^2^(*F*_o_^2^) + (0.077*P*)^2^ + 0.7994*P*] where *P* = (*F*_o_^2^ + 2*F*_c_^2^)/3
2691 reflections	(Δ/σ)_max_ < 0.001
220 parameters	Δρ_max_ = 0.31 e Å^−^^3^
0 restraints	Δρ_min_ = −0.44 e Å^−^^3^

### Special details

**Table:**

Experimental. The crystal was placed in the cold stream of an Oxford Cryosystems Cobra open-flow nitrogen cryostat (Cosier & Glazer, 1986) operating at 100.0 (1) K.
Geometry. All e.s.d.'s (except the e.s.d. in the dihedral angle between two l.s. planes) are estimated using the full covariance matrix. The cell e.s.d.'s are taken into account individually in the estimation of e.s.d.'s in distances, angles and torsion angles; correlations between e.s.d.'s in cell parameters are only used when they are defined by crystal symmetry. An approximate (isotropic) treatment of cell e.s.d.'s is used for estimating e.s.d.'s involving l.s. planes.
Refinement. Refinement of *F*^2^ against ALL reflections. The weighted *R*-factor *wR* and goodness of fit *S* are based on *F*^2^, conventional *R*-factors *R* are based on *F*, with *F* set to zero for negative *F*^2^. The threshold expression of *F*^2^ > σ(*F*^2^) is used only for calculating *R*-factors(gt) *etc*. and is not relevant to the choice of reflections for refinement. *R*-factors based on *F*^2^ are statistically about twice as large as those based on *F*, and *R*- factors based on ALL data will be even larger.

### Fractional atomic coordinates and isotropic or equivalent isotropic displacement parameters (Å^2^)

**Table:**

	*x*	*y*	*z*	*U*_iso_*/*U*_eq_	
O1	0.9767 (3)	0.4254 (2)	0.05460 (11)	0.0420 (6)	
O2	1.2362 (4)	0.3517 (3)	0.10215 (15)	0.0633 (8)	
H1O2	1.1724	0.3988	0.0830	0.095*	
O3	0.5983 (4)	0.4271 (2)	0.41250 (10)	0.0413 (6)	
O4	0.6697 (4)	0.3378 (2)	0.52946 (11)	0.0552 (8)	
H1O4	0.6102	0.4000	0.5273	0.083*	
C1	0.6196 (4)	0.2200 (3)	0.09423 (15)	0.0360 (8)	
H1A	0.5759	0.1550	0.0665	0.043*	
H1B	0.5242	0.2658	0.1110	0.043*	
C2	0.7243 (4)	0.3092 (3)	0.05327 (15)	0.0379 (8)	
H2A	0.7218	0.2792	0.0094	0.045*	
H2B	0.6696	0.3900	0.0537	0.045*	
C3	0.9026 (4)	0.3276 (3)	0.07172 (13)	0.0294 (7)	
C4	0.9885 (3)	0.2325 (3)	0.10845 (12)	0.0221 (5)	
C5	0.8927 (4)	0.1186 (3)	0.13108 (13)	0.0230 (6)	
C6	0.9782 (4)	0.0532 (3)	0.18765 (13)	0.0301 (7)	
H6A	0.9239	−0.0267	0.1947	0.036*	
H6B	1.0944	0.0370	0.1765	0.036*	
C7	0.9731 (4)	0.1276 (3)	0.24997 (14)	0.0272 (6)	
H7A	1.0337	0.2054	0.2446	0.033*	
H7B	1.0269	0.0805	0.2840	0.033*	
C8	0.7911 (4)	0.1547 (2)	0.26801 (13)	0.0226 (6)	
H8A	0.7344	0.0737	0.2708	0.027*	
C9	0.6987 (3)	0.2298 (2)	0.21580 (13)	0.0200 (5)	
C10	0.7108 (4)	0.1569 (3)	0.15117 (13)	0.0241 (6)	
H10A	0.6510	0.0783	0.1585	0.029*	
C11	0.5122 (4)	0.2385 (3)	0.23683 (14)	0.0318 (7)	
H11A	0.4514	0.2889	0.2059	0.038*	
H11B	0.4636	0.1555	0.2363	0.038*	
C12	0.4884 (4)	0.2955 (3)	0.30434 (14)	0.0338 (7)	
H12A	0.5200	0.3828	0.3030	0.041*	
H12B	0.3705	0.2912	0.3157	0.041*	
C13	0.5915 (4)	0.2302 (3)	0.35673 (14)	0.0276 (6)	
C14	0.7769 (4)	0.2146 (2)	0.33451 (13)	0.0227 (6)	
H14A	0.8305	0.2958	0.3336	0.027*	
H14B	0.8364	0.1633	0.3654	0.027*	
C15	0.6017 (4)	0.3139 (3)	0.41616 (14)	0.0290 (6)	
C16	0.6242 (5)	0.2539 (3)	0.48109 (14)	0.0394 (8)	
H16A	0.5201	0.2133	0.4932	0.047*	
H16B	0.7100	0.1900	0.4778	0.047*	
C17	0.5129 (5)	0.1042 (3)	0.37320 (18)	0.0463 (9)	
H17A	0.4050	0.1172	0.3926	0.070*	
H17B	0.5844	0.0603	0.4026	0.070*	
H17C	0.4998	0.0559	0.3347	0.070*	
C18	1.1538 (4)	0.2500 (3)	0.11951 (15)	0.0342 (7)	
H18A	1.2130	0.1872	0.1404	0.041*	
C19	0.8839 (5)	0.0238 (3)	0.07522 (15)	0.0352 (7)	
H19A	0.9938	−0.0099	0.0674	0.053*	
H19B	0.8441	0.0649	0.0372	0.053*	
H19C	0.8085	−0.0427	0.0864	0.053*	
C20	0.7677 (4)	0.3630 (2)	0.21044 (13)	0.0236 (6)	
H20A	0.7370	0.4095	0.2480	0.035*	
H20B	0.7213	0.4026	0.1731	0.035*	
H20C	0.8878	0.3602	0.2068	0.035*	

### Atomic displacement parameters (Å^2^)

**Table:**

	*U*^11^	*U*^22^	*U*^33^	*U*^12^	*U*^13^	*U*^23^
O1	0.0564 (16)	0.0328 (12)	0.0369 (12)	0.0036 (12)	0.0098 (12)	0.0126 (10)
O2	0.0435 (16)	0.0705 (19)	0.076 (2)	−0.0151 (15)	0.0071 (15)	0.0049 (17)
O3	0.0643 (16)	0.0281 (11)	0.0314 (11)	0.0050 (12)	−0.0030 (12)	−0.0080 (10)
O4	0.088 (2)	0.0437 (14)	0.0337 (12)	0.0225 (15)	−0.0180 (13)	−0.0096 (11)
C1	0.0222 (14)	0.056 (2)	0.0301 (15)	0.0036 (14)	−0.0107 (12)	−0.0167 (15)
C2	0.0410 (19)	0.0438 (19)	0.0290 (16)	0.0139 (16)	−0.0147 (14)	−0.0040 (14)
C3	0.0375 (16)	0.0331 (16)	0.0177 (13)	0.0076 (14)	0.0011 (12)	0.0008 (12)
C4	0.0231 (13)	0.0252 (13)	0.0180 (12)	0.0007 (11)	−0.0014 (10)	−0.0005 (10)
C5	0.0233 (13)	0.0212 (13)	0.0245 (13)	0.0021 (11)	−0.0020 (11)	−0.0020 (10)
C6	0.0374 (17)	0.0228 (14)	0.0303 (14)	0.0123 (13)	0.0022 (13)	0.0022 (12)
C7	0.0310 (15)	0.0272 (14)	0.0232 (12)	0.0127 (13)	−0.0015 (12)	0.0032 (11)
C8	0.0289 (14)	0.0133 (11)	0.0254 (13)	0.0001 (11)	0.0041 (11)	0.0011 (10)
C9	0.0154 (11)	0.0198 (12)	0.0248 (13)	−0.0013 (10)	−0.0024 (10)	−0.0051 (11)
C10	0.0212 (13)	0.0241 (14)	0.0269 (14)	−0.0030 (11)	−0.0016 (11)	−0.0068 (12)
C11	0.0190 (13)	0.0440 (18)	0.0325 (16)	−0.0016 (13)	−0.0016 (11)	−0.0148 (14)
C12	0.0187 (14)	0.0483 (18)	0.0345 (15)	0.0004 (14)	0.0012 (12)	−0.0138 (14)
C13	0.0288 (14)	0.0250 (14)	0.0290 (14)	−0.0062 (13)	0.0068 (12)	−0.0070 (12)
C14	0.0293 (14)	0.0145 (12)	0.0242 (13)	0.0027 (11)	0.0002 (11)	0.0009 (10)
C15	0.0265 (14)	0.0320 (15)	0.0286 (15)	0.0014 (13)	0.0044 (12)	−0.0051 (12)
C16	0.050 (2)	0.0347 (17)	0.0333 (18)	0.0056 (17)	0.0005 (15)	−0.0039 (15)
C17	0.057 (2)	0.0367 (18)	0.0451 (19)	−0.0219 (17)	0.0195 (18)	−0.0115 (15)
C18	0.0254 (14)	0.0401 (18)	0.0372 (17)	−0.0031 (14)	−0.0048 (13)	0.0034 (15)
C19	0.0434 (19)	0.0287 (15)	0.0335 (16)	0.0018 (15)	0.0058 (15)	−0.0086 (13)
C20	0.0285 (14)	0.0170 (12)	0.0253 (13)	0.0036 (11)	−0.0081 (11)	−0.0002 (11)

### Geometric parameters (Å, °)

**Table:**

O1—C3	1.254 (4)	C9—C20	1.534 (4)
O2—C18	1.323 (4)	C9—C11	1.551 (4)
O2—H1O2	0.8200	C9—C10	1.560 (4)
O3—C15	1.216 (4)	C10—H10A	0.9800
O4—C16	1.398 (4)	C11—C12	1.545 (4)
O4—H1O4	0.8200	C11—H11A	0.9700
C1—C2	1.529 (5)	C11—H11B	0.9700
C1—C10	1.547 (4)	C12—C13	1.535 (4)
C1—H1A	0.9700	C12—H12A	0.9700
C1—H1B	0.9700	C12—H12B	0.9700
C2—C3	1.484 (5)	C13—C17	1.527 (4)
C2—H2A	0.9700	C13—C15	1.531 (4)
C2—H2B	0.9700	C13—C14	1.556 (4)
C3—C4	1.446 (4)	C14—H14A	0.9700
C4—C18	1.350 (4)	C14—H14B	0.9700
C4—C5	1.515 (4)	C15—C16	1.508 (4)
C5—C6	1.531 (4)	C16—H16A	0.9700
C5—C19	1.547 (4)	C16—H16B	0.9700
C5—C10	1.563 (4)	C17—H17A	0.9600
C6—C7	1.525 (4)	C17—H17B	0.9600
C6—H6A	0.9700	C17—H17C	0.9600
C6—H6B	0.9700	C18—H18A	0.9300
C7—C8	1.525 (4)	C19—H19A	0.9600
C7—H7A	0.9700	C19—H19B	0.9600
C7—H7B	0.9700	C19—H19C	0.9600
C8—C14	1.531 (4)	C20—H20A	0.9600
C8—C9	1.540 (4)	C20—H20B	0.9600
C8—H8A	0.9800	C20—H20C	0.9600
			
C18—O2—H1O2	109.5	C5—C10—H10A	105.5
C16—O4—H1O4	109.5	C12—C11—C9	113.5 (2)
C2—C1—C10	116.4 (3)	C12—C11—H11A	108.9
C2—C1—H1A	108.2	C9—C11—H11A	108.9
C10—C1—H1A	108.2	C12—C11—H11B	108.9
C2—C1—H1B	108.2	C9—C11—H11B	108.9
C10—C1—H1B	108.2	H11A—C11—H11B	107.7
H1A—C1—H1B	107.3	C13—C12—C11	113.7 (3)
C3—C2—C1	117.4 (3)	C13—C12—H12A	108.8
C3—C2—H2A	107.9	C11—C12—H12A	108.8
C1—C2—H2A	107.9	C13—C12—H12B	108.8
C3—C2—H2B	107.9	C11—C12—H12B	108.8
C1—C2—H2B	107.9	H12A—C12—H12B	107.7
H2A—C2—H2B	107.2	C17—C13—C15	111.0 (2)
O1—C3—C4	121.1 (3)	C17—C13—C12	110.1 (3)
O1—C3—C2	119.2 (3)	C15—C13—C12	109.7 (2)
C4—C3—C2	119.7 (3)	C17—C13—C14	111.2 (3)
C18—C4—C3	117.0 (3)	C15—C13—C14	104.7 (2)
C18—C4—C5	123.4 (3)	C12—C13—C14	110.2 (2)
C3—C4—C5	119.6 (3)	C8—C14—C13	112.6 (2)
C4—C5—C6	112.6 (2)	C8—C14—H14A	109.1
C4—C5—C19	108.5 (2)	C13—C14—H14A	109.1
C6—C5—C19	107.4 (2)	C8—C14—H14B	109.1
C4—C5—C10	109.8 (2)	C13—C14—H14B	109.1
C6—C5—C10	109.0 (2)	H14A—C14—H14B	107.8
C19—C5—C10	109.4 (2)	O3—C15—C16	118.9 (3)
C7—C6—C5	113.8 (2)	O3—C15—C13	122.2 (3)
C7—C6—H6A	108.8	C16—C15—C13	118.8 (3)
C5—C6—H6A	108.8	O4—C16—C15	113.8 (3)
C7—C6—H6B	108.8	O4—C16—H16A	108.8
C5—C6—H6B	108.8	C15—C16—H16A	108.8
H6A—C6—H6B	107.7	O4—C16—H16B	108.8
C6—C7—C8	109.5 (3)	C15—C16—H16B	108.8
C6—C7—H7A	109.8	H16A—C16—H16B	107.7
C8—C7—H7A	109.8	C13—C17—H17A	109.5
C6—C7—H7B	109.8	C13—C17—H17B	109.5
C8—C7—H7B	109.8	H17A—C17—H17B	109.5
H7A—C7—H7B	108.2	C13—C17—H17C	109.5
C7—C8—C14	111.9 (2)	H17A—C17—H17C	109.5
C7—C8—C9	112.3 (2)	H17B—C17—H17C	109.5
C14—C8—C9	112.6 (2)	O2—C18—C4	123.4 (3)
C7—C8—H8A	106.5	O2—C18—H18A	118.3
C14—C8—H8A	106.5	C4—C18—H18A	118.3
C9—C8—H8A	106.5	C5—C19—H19A	109.5
C20—C9—C8	111.5 (2)	C5—C19—H19B	109.5
C20—C9—C11	107.9 (2)	H19A—C19—H19B	109.5
C8—C9—C11	106.8 (2)	C5—C19—H19C	109.5
C20—C9—C10	112.4 (2)	H19A—C19—H19C	109.5
C8—C9—C10	108.6 (2)	H19B—C19—H19C	109.5
C11—C9—C10	109.5 (2)	C9—C20—H20A	109.5
C1—C10—C9	114.5 (2)	C9—C20—H20B	109.5
C1—C10—C5	110.2 (2)	H20A—C20—H20B	109.5
C9—C10—C5	114.9 (2)	C9—C20—H20C	109.5
C1—C10—H10A	105.5	H20A—C20—H20C	109.5
C9—C10—H10A	105.5	H20B—C20—H20C	109.5
			
C10—C1—C2—C3	−0.2 (4)	C8—C9—C10—C5	−52.4 (3)
C1—C2—C3—O1	−157.0 (3)	C11—C9—C10—C5	−168.7 (3)
C1—C2—C3—C4	23.7 (4)	C4—C5—C10—C1	57.8 (3)
O1—C3—C4—C18	−4.4 (4)	C6—C5—C10—C1	−178.4 (2)
C2—C3—C4—C18	175.0 (3)	C19—C5—C10—C1	−61.2 (3)
O1—C3—C4—C5	177.0 (2)	C4—C5—C10—C9	−73.3 (3)
C2—C3—C4—C5	−3.6 (4)	C6—C5—C10—C9	50.6 (3)
C18—C4—C5—C6	22.3 (4)	C19—C5—C10—C9	167.7 (2)
C3—C4—C5—C6	−159.2 (2)	C20—C9—C11—C12	−64.2 (3)
C18—C4—C5—C19	−96.4 (3)	C8—C9—C11—C12	55.8 (3)
C3—C4—C5—C19	82.1 (3)	C10—C9—C11—C12	173.2 (3)
C18—C4—C5—C10	144.0 (3)	C9—C11—C12—C13	−53.6 (4)
C3—C4—C5—C10	−37.5 (3)	C11—C12—C13—C17	−74.3 (3)
C4—C5—C6—C7	69.2 (3)	C11—C12—C13—C15	163.3 (3)
C19—C5—C6—C7	−171.3 (3)	C11—C12—C13—C14	48.6 (3)
C10—C5—C6—C7	−52.9 (3)	C7—C8—C14—C13	−174.0 (2)
C5—C6—C7—C8	58.1 (3)	C9—C8—C14—C13	58.3 (3)
C6—C7—C8—C14	172.7 (2)	C17—C13—C14—C8	71.3 (3)
C6—C7—C8—C9	−59.5 (3)	C15—C13—C14—C8	−168.8 (2)
C7—C8—C9—C20	−68.1 (3)	C12—C13—C14—C8	−51.0 (3)
C14—C8—C9—C20	59.3 (3)	C17—C13—C15—O3	−151.8 (4)
C7—C8—C9—C11	174.2 (2)	C12—C13—C15—O3	−30.0 (4)
C14—C8—C9—C11	−58.4 (3)	C14—C13—C15—O3	88.2 (4)
C7—C8—C9—C10	56.2 (3)	C17—C13—C15—C16	31.0 (4)
C14—C8—C9—C10	−176.4 (2)	C12—C13—C15—C16	152.8 (3)
C2—C1—C10—C9	91.3 (3)	C14—C13—C15—C16	−89.0 (3)
C2—C1—C10—C5	−40.0 (3)	O3—C15—C16—O4	−11.1 (5)
C20—C9—C10—C1	−57.5 (3)	C13—C15—C16—O4	166.3 (3)
C8—C9—C10—C1	178.7 (2)	C3—C4—C18—O2	4.4 (5)
C11—C9—C10—C1	62.4 (3)	C5—C4—C18—O2	−177.1 (3)
C20—C9—C10—C5	71.4 (3)		

### Hydrogen-bond geometry (Å, °)

**Table:**

*D*—H···*A*	*D*—H	H···*A*	*D*···*A*	*D*—H···*A*
O2—H1O2···O1	0.82	1.69	2.424 (4)	148
O4—H1O4···O1^i^	0.82	2.07	2.841 (3)	156
C1—H1B···O2^ii^	0.97	2.48	3.368 (5)	152
C12—H12A···O3	0.97	2.41	2.799 (4)	103
C17—H17A···O4^iii^	0.96	2.53	3.460 (5)	164

Symmetry codes:  (i) −*x*+3/2, −*y*+1, *z*+1/2; (ii) *x*−1, *y*, *z*; (iii) *x*−1/2, −*y*+1/2, −*z*+1.

## References

[bb1] Allen, F. H., Kennard, O., Watson, D. G., Brammer, L., Orpen, A. G. & Taylor, R. (1987). *J. Chem. Soc. Perkin Trans. 2*, pp. S1–19.

[bb2] Bernstein, J., Davis, R. E., Shimoni, L. & Chang, N.-L. (1995). *Angew. Chem. Int. Ed. Engl.* **34**, 1555–1573.

[bb3] Bruker (2005). *APEX2*, *SAINT* and *SADABS* Bruker AXS Inc., Madison, Wisconsin, USA.

[bb4] Chantrapromma, S., Fun, H.-K., Pakhathirathien, C., Karalai, C. & Chantrapromma, K. (2007). *Acta Cryst.* E**63**, o459–o461. 10.1107/S1600536812002565 PMC3275262 22347118

[bb5] Cosier, J. & Glazer, A. M. (1986). *J. Appl. Cryst.* **19**, 105–107.

[bb6] Cremer, D. & Pople, J. A. (1975). *J. Am. Chem. Soc.* **97**, 1354–1358.

[bb7] Fun, H.-K., Pakhathirathien, C., Chantrapromma, S., Karalai, C. & Chantrapromma, K. (2006). *Acta Cryst.* E**62**, o5539–o5541. 10.1107/S1600536812002565 PMC3275262 22347118

[bb8] Hu, W.-M., Li, M.-Y., Li, J., Xiao, Q., Feng, G. & Wu, J. (2010). *J. Nat. Prod* In the press. doi:10.1021/np100484w. 10.1021/np100484w 20886837

[bb9] Sheldrick, G. M. (2008). *Acta Cryst.* A**64**, 112–122. 10.1107/S0108767307043930 18156677

[bb10] Spek, A. L. (2009). *Acta Cryst.* D**65**, 148–155. 10.1107/S090744490804362X PMC2631630 19171970

[bb11] Zhang, Y., Deng, Z., Gao, T., Proksch, P. & Lin, W. (2005). *Phytochemistry*, **66**, 1465–1471. 10.1016/j.phytochem.2005.04.018 15927216

